# An overview of the efficacy and signaling pathways activated by stem cell-derived extracellular vesicles in diabetic kidney disease

**DOI:** 10.3389/fendo.2022.962635

**Published:** 2022-07-28

**Authors:** Yongda Lin, Qian Yang, Jiali Wang, Xiutian Chen, Yiping Liu, Tianbiao Zhou

**Affiliations:** Department of Nephrology, Second Affiliated Hospital, Shantou University Medical College, Shantou, China

**Keywords:** diabetic kidney disease, mesenchymal stem cells, extracellular vesicles, signaling pathway, end-stage renal disease

## Abstract

Diabetic kidney disease (DKD) is one of complications of diabetes mellitus with severe microvascular lesion and the most common cause of end-stage chronic kidney disease (ESRD). Controlling serum glucose remains the primary approach to preventing and slowing the progression of DKD. Despite considerable efforts to control diabetes, people with diabetes develop not only DKD but also ESRD. The pathogenesis of DKD is very complex, and current studies indicate that mesenchymal stromal cells (MSCs) regulate complex disease processes by promoting pro-regenerative mechanisms and inhibiting multiple pathogenic pathways. Extracellular vesicles (EVs) are products of MSCs. Current data indicate that MSC-EVs-based interventions not only protect renal cells, including renal tubular epithelial cells, podocytes and mesangial cells, but also improve renal function and reduce damage in diabetic animals. As an increasing number of clinical studies have confirmed, MSC-EVs may be an effective way to treat DKD. This review explores the potential efficacy and signaling pathways of MSC-EVs in the treatment of DKD.

## Introduction

The global prevalence of diabetes is projected to rise from 9.3% in 2019 to 10.9% in 2045 (1). Diabetic kidney disease (DKD) is a serious complication caused by diabetes and occurs in 20-40% of people with diabetes (2, 3). Treatment of DKD has included blood pressure control with angiotensin receptor blockers or angiotensin-converting enzyme inhibitors and strict glycemic control (4). However, many patients still progress to the stage of end-stage renal disease (ESRD) (5). From 2000 to 2015, DKD as a percentage of chronic kidney disease increased from 22.1% to 31.3% (6) and is the most common cause of ESRD in many developed countries. When compared with other diabetic complications, the prevalence of DKD disease has not notably reduced over the past 20 years (7). There is an urgent need to develop effective therapeutic strategies to preserve the renal function and slow the progression of DKD.

Mesenchymal stromal cells (MSCs) are a group of cells with differentiation and proliferative potential (8). When treated with appropriate compounds, they can differentiate into cells of all mesodermal lineages, such as fibroblasts, myocytes, adipocytes, osteocytes, or chondrocytes (9, 10). Studies have shown that miR-124a can stimulate the differentiation of mesenchymal stem cells sourced bone marrow into islet-like cells and thus alleviate DKD (11). MSCs mainly used for DKD include adipose-derived (AD-MSCs), umbilical cord-derived (UC-MSCs), bone marrow-derived (BM-MSCs), and human urine-derived (HU-MSCs). MSCs protect the kidney in two ways. One is homing and differentiation, where MSCs recognize damaged tissue and then home and integrate into specific sites, and the other is through paracrine action. However, the main problem after direct administration of MSCs is that they do not target the target tissue. The infusion of MSCs *via* direct acute infusion or coupled with implanted continuous pumps directly into the kidney is the main way (12). After MSCs are injected into the tail vein of rats, most of the stem cells appear in the lungs (13). To determine whether MSCs exist in rat tissues, DNA was extracted from rat organs and a human Alu sequence was detected. Human Alu sequences were detected in the peritubular region, lung, and spleen in rats within 24 hours after injection of labeled MSCs, but rarely in the glomerulus and pancreas (14). Implanted continuous pumps directly into the kidney have the advantage of local tissue effect, but it is difficult to implement and difficult to popularize clinically. As only minimal numbers of donor MSCs are detected in the renal tissue, the therapeutic effect of MSCs on kidney injury appears to be attributable to lots of paracrine factors. These factors facilitate the renal repair by paracrine-mediated actions, including cell-cell interactions reactivating endogenous repair systems, and the release of extracellular vesicles (EVs) (15). This review explores the potential efficacy and signaling pathways of MSC-EVs in the treatment of DKD.

## Characteristics of stem cell products

**Figure 1:**
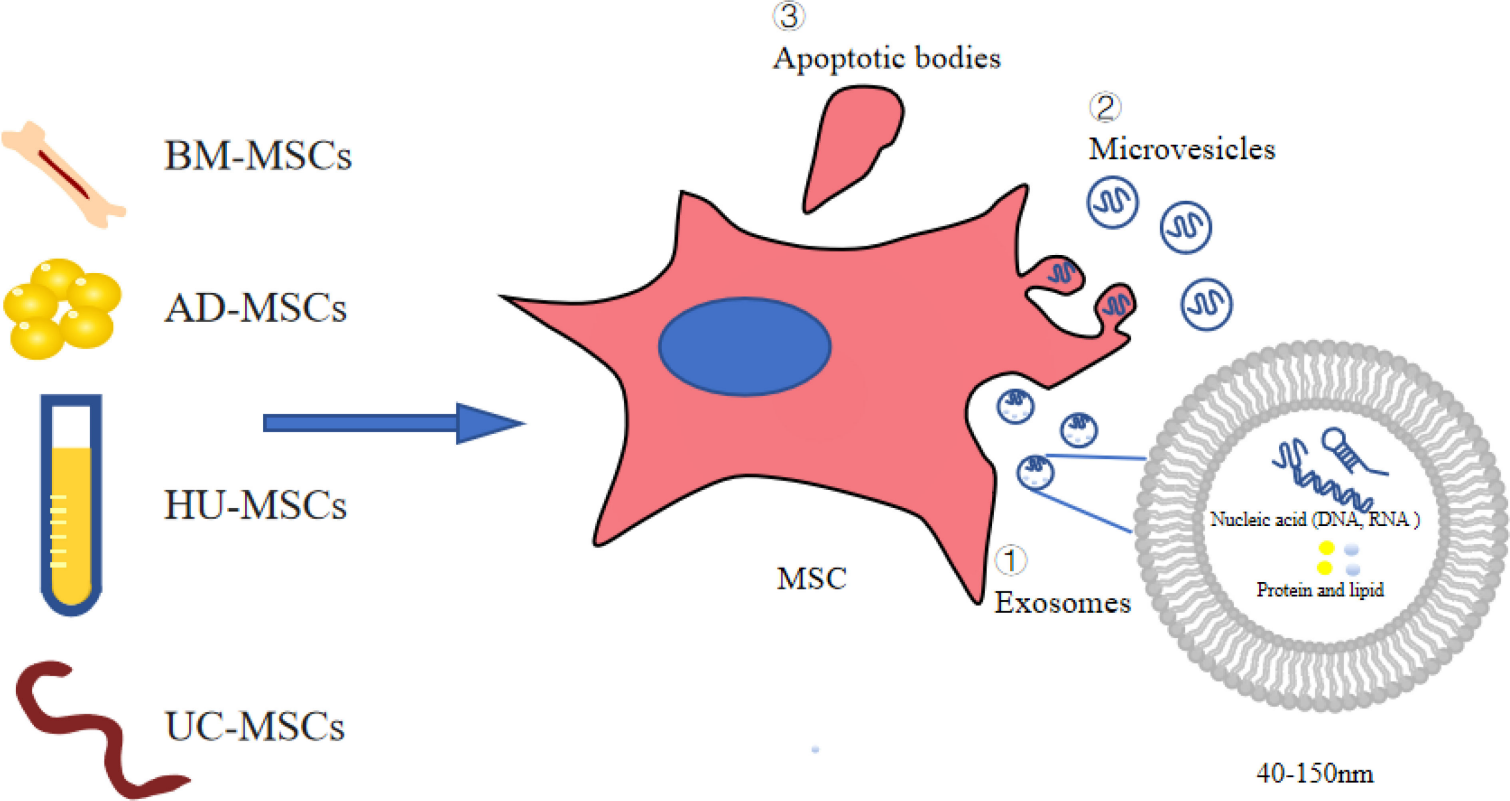
Extracellular vesicles derived from stem cells. AD-MSCs, adipose-derived MSCs; BM-MSCs, bone marrow-derived MSCs; UC-MSCs, umbilical cord-derived MSCs; HU-MSCs, human urine-derived MSCs; MSCs. mesenchymal stromal cells.

MSC-conditioned medium (MSC-CM) is rich in EVs secreted by MSCs. As stem cell products, EVs are generally classified into three categories: apoptotic bodies, microvesicles (MVs), and exosomes (Exos), which vary in size, origin, and release mechanism (16, 17). MVs are 50 nm-1000 nm in diameter and are shed directly from the cytoplasmic membrane (16, 18, 19). Their release is initiated by budding outward from the membrane surface (16, 19). Apoptotic bodies are released during apoptosis. The diameter of apoptotic vesicles is reported to range between 1000 nm and 5000 nm (20). The reported diameter of Exos is between 40 and 150 nm (21). Exos carry complex molecular cargoes such as proteins, lipids and nucleic acids (e.g., DNA, miRNA, circRNA) (**Figure 1**). A series of studies have shown that stem cell Exos protect the kidney from damage through multiple pathways involving anti-apoptotic, anti-inflammatory, anti-oxidative, anti-fibrotic roles and regulate podocyte autophagy (22, 23).

## Amelioration of podocyte injury by stem cell products

Podocytes, important intrinsic cells of the glomerulus, are involved in protein filtration in the glomerulus and play an important role in the maintenance of kidney function (23, 24). Podocyte injury is an important characteristic of DKD. Podocyte loss contributes to the development of DKD (25). MSC-EVs are found that they can protect the podocytes in DKD in multiple ways, including inhibition of apoptosis and fibrosis and enhancement of autophagy, all perhaps due to the presence of a large number of growth factors and miRNAs in MSC-EVs (26).

High glucose (HG)-induced podocyte damage *in vitro* can mimic the state of podocytes in patients with DKD. Jiang et al. (27) conducted a study on human podocytes, and reported that HU-MSC-EVs reduced HG-induced podocyte apoptosis *in vitro*. UC-MSCs secrete EVs carrying bone morphogenetic protein-7 (BMP-7) and high levels of vascular endothelial growth factor (VEGF) (**Table 1**). The activation of cytokines such as BMP-7 or VEGF is important for the survival of podocyte. VEGFA is highly expressed in the podocytes and is required to maintain endothelial cell function (46). However, VEGFA is overexpressed in the early stage of DN, and blocking VEGFA reduces proteinuria in DN (47). Duan et al. (28) showed that silencing of VEGF attenuates podocyte inflammation and reduces apoptosis. Moreover, miR-16-5p encapsulated within HU-MSCs can inhibit VEGFA expression in podocytes induced by HG, promote podocyte viability and decrease the rate of apoptosis (**Table 1**). AD-MSC-EVs can transfer miR-26a-5p to mouse podocytes *in vitro* to inhibit podocyte apoptosis by downregulating nuclear factor kappa-B (NF-κB)/VEGFA and toll-like receptor 4 (TLR4) signaling pathways (29). Pyruvate dehydrogenase kinase 4 (PDK4) is a molecular target of miR-15b-5p (48). Zhao et al. (30) reported that AD-MSC-CM miR-15b-5p directly binds to PDK4 in podocytes from mouse and inhibits the expression of PDK4 mRNA and protein. Inhibition of PDK4 can reduce the activation of VEGFA and downregulate the inflammation and cell apoptosis (**Table 1**). The results of several studies showed that stem cell products inhibit VEGFA expression through multiple pathways, thereby attenuating HG-induced damage to podocytes.

**Table 1:** Characteristics of assessing the efficacy of MSC products for DKD *in vitro*.

Author	Cell model	Stem cell type	Treatment effect
	Podocyte		
Jiang et al. (27)	HPDCs	USC-EVs	Reduce podocyte apoptosis (BMP-7, VEGF, TGF-β and angiogenin↑)
Duan et al. (28)	HPDCs	HUC-EVs	Increase podocyte viability and reduce rate of apoptosis (miR-16-5p↑→ VEGFA↓)
Duan et al. (29)	MPC5	ASC-EVs	Inhibit podocyte apoptosis (miR-26a-5p↑→ TLR4↓→ NF-κB/VEGFA↓)
Zhao et al. (30)	MPC5	ASC-CM	Inhibit podocyte apoptosis and inflammation (miR-15b-5p↑→ PDK4↓→ VEGFA↓)
Li et al. (31)	MPC5	ASC-CM	Reduce podocyte apoptosis (EGF↑)
Zhang et al. (14)	MPC5	ASC-CM	Reduce podocyte apoptosis (GDNF↑)
Jin et al. (32)	MPC5	ASC-EVs	Promote autophagy and inhibit podocyte apoptosis (miR-486↑→ Smad1↓→ mTOR↓)
Jin et al. (33)	MPC5	ASC-EVs	Attenuate EMT of Podocytes (miR-215-5p↑→ ZEB2↓)
	Mesangial cells		
Li et al. (34)	SV40-MES-13	USC-CM	Alleviate Fibrosis (MAPK↓ and PI3K/Akt↓→ MMP2 and MMP9↑)
Lv et al. (35)	HBZY-1	BMSC-CM	Inhibit fibrosis and oxidative stress and reduce the expression of GLUT1
Bai et al. (36)	HBZY-1	BMSC-CM	Inhibit fibrosis and inflammation (LXA4↑→ TGF-β↓)
Gallo et al. (37)	MCs	BMSC-EVs HLSC-EVs	Inhibit fibrosis and interference with mitochondrial dysfunction (miR-222↑/miR-21↓) →, STAT5A↓→, TGF-β↓)
Hao et al. (38)	GMC	ASC-EVs	Inhibit fibrosis and reduce apoptosis (miR-125a↑→ HDAC1/ET1↓)
	Renal tubular epithelial cells		
Nagaishi et al. (39)	PTECs	BMSC-CM	Anti-apoptotic and anti-degenerative (TGF-β1↓, lectin and ZO-1↑)
Park et al. (40)	NRK-52E	USC-CM	Inhibited ECM and EMT (TGF-β1↓)
Zhong et al. (41)	HK-2	HUC-MVs	Reverse EMT by restarting the blocked cell cycle (miR-451a↑→ P15 and P19↓)
Rao et al. (42)	HK-2	SHED-CM BMSC-CM	Inhibit AGE-induced EMT (E-cadherin↑, fibronectin and vimentin↓)
Ali et al. (43)	HK-2	WJMSCs-CM	Attenuate oxidative stress-mediated apoptosis and fibrosis (CHIP↑→ MAPK↓)
Lee et al. (44)	HK-2	USC-CM	Reverse mitochondrial dysfunction (Arg↓→ M1 macrophages↓→ NO, IL-6, TNF- IL-1↓)
Konari et al. (45)	NRK-52E	BMSC-CM	Inhibit apoptosis and reduce ROS production by transferring mitochondria

ASC, adipose-derived MSCs; BMSC, bone marrow-derived MSCs; GMC, rat glomerular mesangial cells; HBZY-1, The rat glomerular mesangial cell line; HK-2, human proximal tubular epithelial; HLSC, human liver stem-like cells; HPDCs, Human podocytes; HUC, human urine-derived MSCs; MCs, Human mesangial cells; MPC5, A mouse podocyte clone 5; NRK-52E, renal tubularduct epithlialcells of rat; PTECs, proximal tubular epithelial cells; SHED, Stem cells from human exfoliated deciduous teeth; SV40-MES-13, mouse mesangial cell; WJMSCs, Wharton’s jelly-derived MSCs; USC, umbilical cord-derived MSCs; AGEs, advanced glycation end products; Arg1, arginase-1; BMP-7, bone morphogenetic protein-7; CHIP, Carboxyl terminus of HSP70 interacting protein; CM, conditioned medium; EGF, epidermal growth factor; EMT, epithelial-mesenchymal transition; ET-1, endothelin-1; GDNF, glial cell-derived neurotrophic growth factor; HDAC1, histone deacetylase 1; HGF, hepatocyte growth factor; LXA4, lipoxin A4; MAPK, mitogen-activated protein kinase; MMP, metalloproteinase; PDK4, Pyruvate dehydrogenase kinase 4; ROS, reactive oxygen species; STAT1, Signal Transducers and Activators of Transcription-1; TLR4, toll-like receptor 4; VEGF, vascular endothelial growth factor; ZEB2,Zinc finger E-box-binding homeobox 2; ZO-1, zona occludens protein-1.

Li et al. (31) reported that AD-MSC-CM reduced podocyte apoptosis induced by HG, downregulated activated caspase-3, increased epithelial growth factor (EGF), and prevented the rearrangement and downregulation of synaptopodin. However, it did not affect the levels of glial cell line-derived neurotrophic growth factor (GDNF), insulin-like growth factor binding protein and placental growth factor (**Table 1**). However, a study pointed out that GDNF promotes mouse podocyte survival *in vitro* and protects mouse podocytes from apoptosis (49). Zhang et al. (14) confirmed this and found that HG can reduce podocytic synaptopodin, and AD-MSC-CM can increase synaptopodin expression in podocytes. After blocking GDNF in AD-MSC-CM with GDNF-NtAb, the therapeutic effect of podocytic synaptopodin was partly abolished (**Table 1**). These studies demonstrated that stem cell products reduce podocytic apoptosis by modulating cytokines.

Autophagy is a lysosomal degradation pathway in cells for maintaining cellular homeostasis and cellular health under various stress conditions (50). Evidence suggests that podocytes have high levels of basal autophagy, which may be a mechanism to maintain cellular homeostasis (51). Jin et al. (32) showed that AD-MSC-EVs can inhibit p-mTOR/mTOR, Smad1, p62, and apoptosis, but they can increase Beclin1 and LC3 (**Table 1**). AD-MSC-EVs ameliorate podocyte damage by inhibiting the miR-486/Smad1/mTOR signaling pathway. This suggested that stem cell products can regulate the autophagy of podocytes.

HG may induce podocyte epithelial-mesenchymal transition (EMT) through multiple pathways (52). Jin et al. (33) showed that, with podocyte dysfunction, several EMT-related miRNAs, including miR-3066-5p, miR-879-5p, miR-251-5p, and miR-7a-5p, were increased by the addition of AD-MSC-EVs (**Table 1**). Potentially, AD-MSC-EVs can mediate the shuttling of miR-215-5p to podocytes, possibly through inhibiting the transcription of zinc finger E-box-binding homeobox 2 (ZEB2), thereby attenuating EMT of podocytes.

## Stem cell products ameliorate fibrosis in mesangial cells

The signaling pathway of TGF-β can play an important role in the fibrogenesis, especially in DKD renal fibrosis, and can be activated by high glucose (53). Endothelin-1 (ET-1) promotes fibrosis and inflammation in DKD (54). ET-1 and TGF-β1 induce collagen I production by fibroblasts (55). Li et al. (34) found that blocking TGF-β1 by UC-CM inhibited the expression of collagen I and fibronectin in mesangial cells treated with HG. Antifibrotic effects of MSC paracrine in DN may be detected by EVs shed by MSCs. BM-MSC-CM treatment remarkably reduced expressions of TGF-β and TGF-β-induced glucose transporter 1, thus inhibiting fibrosis and oxidative stress. A large amount of hepatocyte growth factor (HGF) was detected in CM, and the effects of CM on TGF-β and TGF-β-induced glucose transporter expression could be blocked by the addition of neutralizing antibodies against HGF (**Table 1**) (35). This indicated that HGF in CM alleviates mesangial cells’ fibrosis and oxidative stress. Co-culture of BM-MSCs and mesangial cells alleviated cell fibrosis by targeting lipoxin A4 (LXA4) to modulate TGF-β/Smad signaling (36). STAT5A was identified as a transcriptional regulator of miR-21, which in turn affects collagen production and TGF-β expression in mesangial cells. MSC-EV-miR-222 regulates STAT5 expression and indirectly regulates TGF-β expression (37). Hao et al. (38) found that AD-MSC-EVs inhibit the histone deacetylase 1 (HDAC1)/ET1 axis by secreting miR-125a and suppressing IL6, collagen I and fibronectin levels in HG-treated mesangial cells. All these results suggested that MSC-EVs alleviate mesangial cell fibrosis by regulating the expression of TGF-β and ET-1 (**Table 1**).

## Stem cell products ameliorate fibrosis in renal tubular epithelial cells

Renal tubulointerstitial fibrosis, characterized by EMT of renal tubular epithelial cells (RTEC), is a major cause of diabetic renal fibrosis (56). Important roles of different tubular responses, such as partial EMT, cell cycle arrest and metabolic defects are involved in renal fibrosis (57). Nagaishi et al. (39) found that MSC-EVs can reduce intracellular adhesion molecule-1 (ICAM-1) and TGF-β1 in RTEC isolated from streptozotocin (STZ)-induced diabetic rats, and zona occludens protein-1 (ZO-1) expression was increased in RTEC cultured with EVs or MSCs. UC-MSC-CM inhibited TGF-β1-induced EMT and extracellular matrix accumulation (ECM) in RTEC (NRK-52E) (**Table 1**) (40). Zhong et al. (41) further found that MSC-EV-miR-451a reverses EMT by inhibiting P15 and P19 to restart the blocked cell cycle (**Table 1**). Inhibition of fibrosis by regulating the cell cycle by miRNA may be a new therapeutic modality.

Advanced glycation end products (AGEs) induce apoptosis and increase the expression of inflammatory and fibrotic genes in renal tubular cells (58). Hyperglycemia-induced reactive oxygen species (ROS) generation activates mitogen-activated protein kinases (MAPKs), which are involved in the EMT of RTEC (HK2) (59, 60). Co-cultured stem cells from human exfoliated deciduous teeth inhibit AGE-induced EMT in HK-2 cells (**Table 1**) (42). Ali et al. (43) reported that the protein expression of Carboxyl terminus of HSP70 interacting protein (CHIP) was reduced under HG treatment, which can limit the therapeutic potential and survivability of Wharton’s jelly-derived MSCs (WJMSCs). CHIP-overexpressing WJMSCs attenuate fibrosis and cell apoptosis mediated by hyperglycemia-induced oxidative stress in HK-2 cells *via* activation of the MAPKs (**Table 1**). This proves that MSC-EVs can inhibit the production of AGEs and ROS and downregulate the fibrosis of renal tubular epithelial cells.

Mitochondria have been recognized as key regulators of inflammation, cell death, metabolism and ROS production (61). M1 macrophages express pro-inflammatory cytokines, and M2 macrophages are thought to regulate inflammatory responses and promote tissue repair (62). Arginase-1 (Arg1) is a marker of M2 macrophages, and co-culture of macrophages with MSCs increases Arg1 and decreases the expression of M1 markers. UC-MSCs reverse mitochondrial function in HK-2 cells by inducing Arg1 in macrophages (44). It should be noted that AD-MSCs cannot reverse mitochondrial dysfunction, which may indicate that the beneficial effect of MSCs is limited to umbilical cord blood. Konari et al. (45) showed that BM-MSCs also transfer their mitochondria to damaged RTEC when co-cultured *in vitro*, inhibiting apoptosis and reducing ROS production of damaged RTEC (**Table 1**).

**Figure 2:**
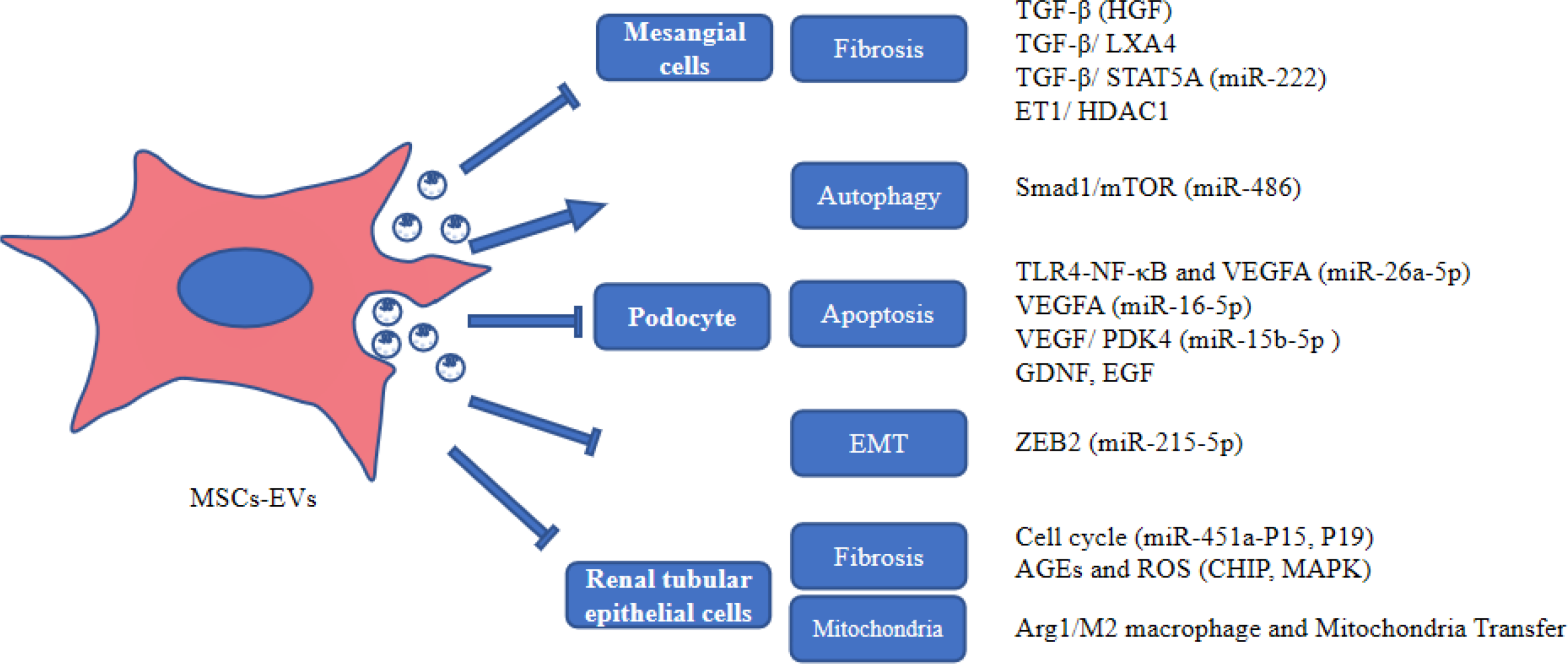
The role of MSC-EVs in protecting kidney cells. AGEs, advanced glycation end products; Arg1, arginase-1; EGF, epidermal growth factor; GDNF, glial cell-derived neurotrophic growth factor; HDAC1, histone deacetylase 1; HGF, hepatocyte growth factor; LXA4, lipoxin A4; MAPK, mitogen-activated protein kinase; PDK4, Pyruvate dehydrogenase kinase 4; ROS, reactive oxygen species; VEGF, vascular endothelial growth factor; ZEB2, Zinc finger E-box-binding homeobox 2.

MSC-EVs protect HG-induced damaged kidney cells through multiple pathways, suggesting that MSC-EVs may be a potential therapeutic modality for DKD (**Figure 2**).

## Efficacy of MSC-EVs for DKD: A preclinical model

Intravenous injection of MSC-EVs can improve renal function and histological damage in diabetic animals. Hyperglycemia is a major factor in the occurrence of DKD. EVs secreted from AD-MSCs, BM-MSCs and UC-MSCs are able to reduce blood glucose. Furthermore, EVs can reduce serum creatinine (SCr), blood urea nitrogen (BUN), urinary protein (URPO), and urine albumin-to-creatinine ratio (UACR), and increase creatinine clearance (CCR) (28, 38, 63). Hyperlipidemia is an important risk factor for vascular complications of chronic kidney disease (64). BM-MSC-EVs reduced total cholesterol (TC) and triglycerides (TG) in a DKD mouse model (65, 66). Jiang et al. (27) found that HU-MSC-EVs could reduce the urinary microalbumin excretion and urine volume of DKD rats.

Duan et al. (29) showed that AD-MSC-EVs notably alleviated the histopathological changes associated with DKD, such as reducing ECM accumulation in their kidney tissues and the thickening of basement membrane. Jiang et al. (27)found that UC-MSC-EVs could prevent cell apoptosis in diabetic rats. Further, UC-MSC-EVs treatment significantly ameliorated mesangial expansion and promoted endothelial cell proliferation from glomerulus in the early stages of impairment in DKD kidney. Ebrahim et al. (63) found that injection of EVs in DKD rats reduced histological damage. EVs alleviated diffuse thickening of glomerular basement membrane and extensive fusion and disappearance of foot process.

Inflammation and fibrosis play a key role in the pathogenesis of DKD. Xiang et al. (67) found that UC-MSC-CM or UC-MSC-EVs suppressed IL-1β, TNF-α, IL-6, and TGF-β in HG-injured human renal glomerular endothelial cell line (hrGECs) and RTECs (HK2 and NRK-52E). Duan et al. (28) reported that HU-MSC-EVs can reduce the expression of VEGFA, monocyte chemoattractant protein-1 (MCP-1), TNF-α and TGF-β1 in diabetic mice. This means that the UC-MSC product can relieve inflammation in diabetic mice. MCP-1 is also known as the C-C motif chemokine ligand 2 (CCL2). CCL2 can stimulate the production of TGF-β1 in mesangial cells and macrophages, and TGF-β1 feeds back to increase the CCL2 expression in mesangial cells (68). Substantial evidence indicates that TGF-β/Smad signaling takes part in the development and progression of fibrosis of kidney (69). Hao et al. (38)found that AD-MSC-EVs reduced the protein expression of the Col-I (fibrosis-related marker) and suppressed mesangial hyperplasia in DKD rats (**Table 2**). Mao et al. (65) found that BM-MSC-EVs miR-let-7a inhibited the expression of N-cadherin and vimentin (**Table 2**). Furthermore, Nagaishi et al. (39) showed that BM-MSC-CM can reduce TNF-α, ICAM-1 and increase ZO-1 (**Table 2**). Zhong et al. (41) showed that UC-MSC-EVs reduced α-SMA, increased E-cadherin and prevented histological damages (**Table 2**). Grange et al. (70) reported that human liver stem-like cells (HLSC)-EVs and BM-MSC-EVs down-regulate genes involved in the pathogenesis of fibrosis, such as tissue inhibitor of metalloproteinases (TIMP), metalloproteinase 3 (MMP3), collagen I, TGF-β and α-SMA, the FAS ligand, CCL3, and Snail (**Table 2**). This indicates that injection of MSC-EVs from various sources can alleviate fibrosis in diabetic animals.

**Table 2:** Characteristics of preclinical studies assessing the efficacy of MSC products for DKD *in vivo*.

Author	Stem cell type	Model	Treatment		Treatment effect
Hao et al. (38)	ASC-EVs	STZ SD	Exos (50μg) *via* tail vein injection	Twice a week for 3 weeks	miR-125a protected DKD in rats through inhibiting the HDAC1/ET1 axis
Mao et al. (65)	BMSC-EVs	STZ SD	Exos (100μg) *via* tail vein injection	Once a week for 12 weeks	miR-let-7a inhibited apoptosis and oxidative stress in renal cells and suppressed the expression of N-cadherin and vimentin
Nagaishi et al. (39)	BMSC-CM	STZ mice	CM (2mg/kg) *via* tail vein injection	Once a day for 8 weeks	Anti-inflammation and inhibition of EMT (TNF-α, p38-MAPK, ICAM-1, TGF-β↓ and ZO-1↑)
Zhong et al. (41)	HUC-EVs	STZ mice	MVs (1.5mg/kg) *via* tail vein injection	Once a week for 8 weeks	miR-451a reduced renal fibrosis through down-regulation of the P15INK4b and P19INK4d
Grange et al. (70)	HLSC-EVs BMSC-EVs	STZ mice	EVs(1×10^10^ particles) *via* tail vein injection	Once a week for 4 weeks	EVs down regulated genes involved in the development of fibrosis (MMP3, collagen I, TIMP, SNAI1, CCL3, Serpina1, interferon γ, Fas Ligand↓)·
Duan et al. (29)	ASC-EVs	C57BL/KsJ db/db	–	Once a week for 12 weeks	miR-26a-5p reduced the pathological symptoms and cell apoptosis
Jiang et al. (27)	USC-EVs	STZ SD	Exos (100μg) *via* tail vein injection	Once a week for 12 weeks	Anti-apoptosis, promoted glomerular endothelial cell proliferation and ameliorated mesangial expansion
Ebrahim et al. (63)	BMSC-EVs	STZ SD	Exos (100μg/kg) *via* tail vein injection	Once a day for 4 weeks	Induction of autophagy through the mTOR signaling pathway

ASC, adipose-derived MSCs; BMSC, bone marrow-derived MSCs; HLSC, human liver stem-like cells; HUC,human urine-derived MSCs; USC, umbilical cord-derived MSCs; CCL3, C-C motif chemokine ligand 3; CM, conditioned medium; EMT, epithelial-mesenchymal transition; ET-1, endothelin-1; EVs, extracellular vesicles; HDAC1, histone deacetylase 1; ICAM-1, intracellular adhesion molecule-1; MAPK, mitogen-activated protein kinase; MVs, microvesicles; MMP3, metalloproteinase 3; TIMP, tissue inhibitor of metalloproteinases; SD, Sprague-Dawley rat; STZ, streptozotocin; ZO-1, zona occludens protein-1.

In addition to being anti-inflammatory and anti-fibrotic, MSC-EVs protect DKD animals by other means. With the effecting by increased oxidative stress and reduced NO bioavailability, endothelial dysfunction is a hallmark feature of type 2 diabetes mellitus and DKD (71). Mao et al. (65) found that BM-MSC-EVs suppress oxidative stress, based on decreases in NO and MDA content, and elevations of GSH-Px and SOD activities. EVs injection can alleviate the oxidative stress reaction in diabetic mice and play a protective role in DKD through down-regulation of USP22. Autophagy, a conserved and important “self-eating” pathway, is an important mechanism for maintaining glomerular and tubular homeostasis, and is involved in various aspects of renal injury, aging and disease (72). Ebrahim et al. (63) reported that BM-MSC-EVs can inhibit mTOR, and S6K1 protein expression, and increase Beclin-1, LC3-I, LC3-II, and p62 protein expression. MSC-EVs ameliorate DKD by modulating the mTOR-related autophagy pathway (**Table 2**). Duan et al. (29) reported that Bcl-2 protein was significantly increased by AD-MSC-EVs, whereas the protein expressions of cleaved caspase-3, caspase-3, and Bax were reduced. MiR-26a-5p produced by AD-MSC-EVs not only reduced podocyte apoptosis *in vitro*, but also reduced apoptosis in spontaneously diabetic mice.

Overall, through multiple studies injected with stem cell products from different sources, we found that stem cell products can alleviate kidney damage in animal models of DKD (**Table 1**).

## Effect of stem cell extracellular vesicles on inflammatory factors and their potential signaling pathways

Phosphorylation of signal transducers and activators of transcription-1 (STAT1) is induced by multiple TLRs (TLR2, TLR4, TLR9). Tumor necrosis factor receptor-associated factor-6 (TRAF6) is a most important factor to activate TLR signaling. After activation of TRAF6, STAT1 is phosphorylated and translocated to the nucleus (73). Zhang et al. (74) found that UC-MSCs-derived miR-146a-5p can target the TRAF6 and significantly decreased the expression of p-STAT1. They identified that miR-146a-5p targeted the signaling pathway of TRAF6-STAT1 to inhibit renal inflammation and restore the function of kidney by promoting the polarization of M2 macrophage. Transcription factors, such as NF-κB, can regulate inflammation, immunological responses and cell proliferation (75). In DKD, the transposition of NF-κB into the nucleus can activate its target genes, including the inflammatory mediators of its downstream, such as nitric oxide synthase, TNF-β1, IL-1 and ICAM-1, which subsequently cause persistent and increased inflammation leading to overexpression of fibronectin and ECM accumulation in mesangial cells (76). Duan et al. (29) reported that AD-MSC-EV-miR-26a-5p alleviated both the ECM accumulation in kidney tissues and the thickening of basement membrane, and inhibited apoptosis in mouse podocytes *in vitro* by inhibiting signaling pathways of NF-κB/VEGFA and TLR4.

Autophagy promotes the degradation of excess or malfunctioning cellular components, including invasive microorganisms, misfolded proteins, damaged organelles and cells themselves (77). In DKD, autophagy represents the cooperation of related gene products of multiple autophagy (23). In general, mTORC1 is regarded as a negative regulator for the autophagy. Enhanced mTORC1 activity was observed in type 1 and type 2 DKD diseases from animal models. Treatment with rapamycin (an inhibitor of mTORC1) can suppress the progression of DKD disease induced by STZ in type 1 and type 2 diabetes from rats or mice (78, 79). Injection of MSC-EVs into the tail vein of DKD rats resulted in significant up-regulation of autophagy-related proteins (LC3II and Beclin-1), and significant down-regulation of mTOR gene expression. The results indicated that BM-MSC-EVs enhance autophagy by inhibiting mTOR (63). Jin et al. (32) found that AD-MSC-EVs inhibited p62/LC3 and mTOR signaling pathways, increased the levels of autophagy-related proteins, and ameliorated cell damage of podocyte by inhibiting the signaling pathway of miR-486/Smad1/mTOR. Cai et al. (80) showed that miR-125b from MSC-EVs could induce the cell autophagy to inhibit HKCs apoptosis induced by HG *via* signaling pathway of Akt. Collectively, multiple miRNAs in EVs inhibited mTORC1 expression by inhibiting AKT and finally upregulated autophagy.

**Figure 3:**
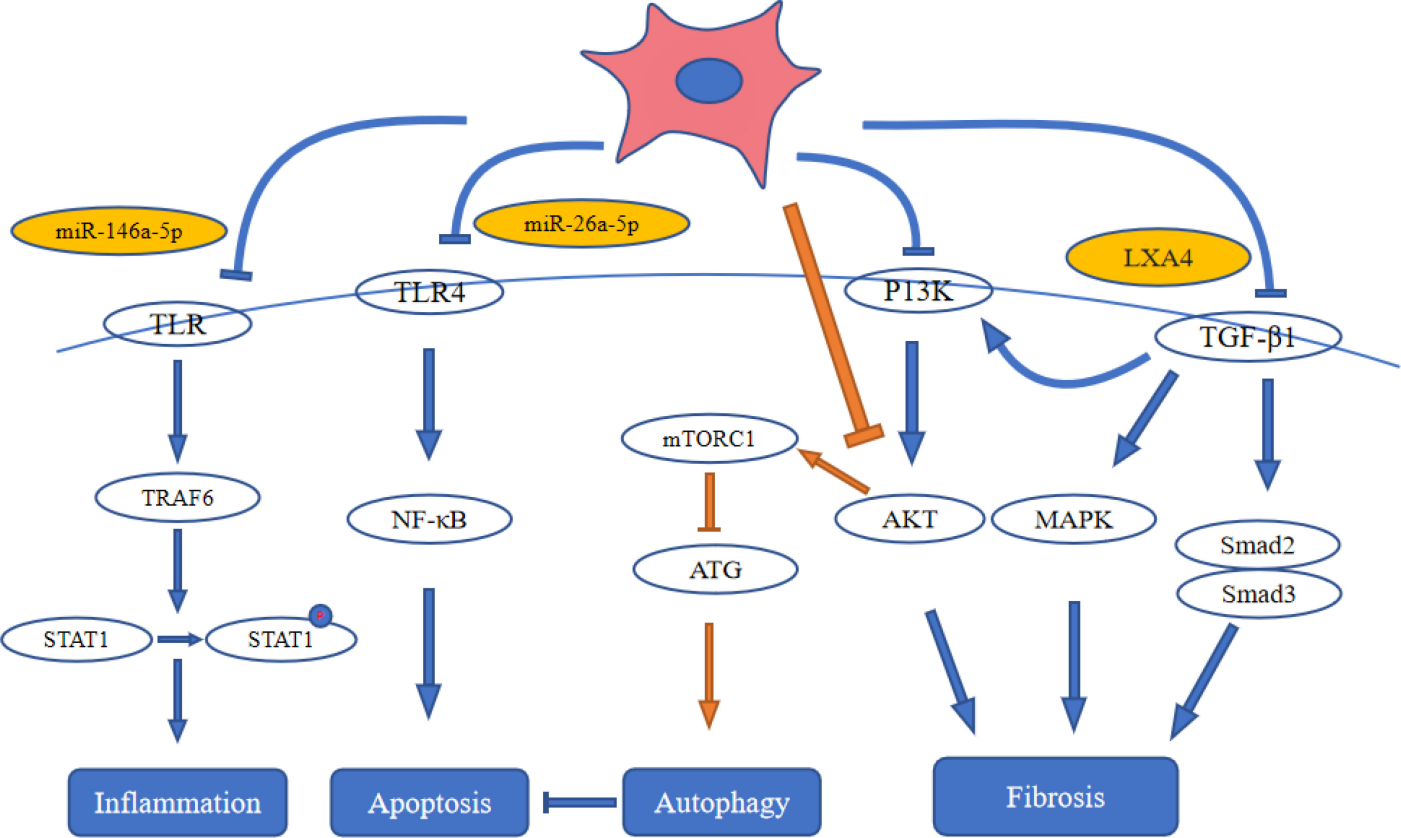
Stem cell therapy influences signal pathways in diabetic kidney disease. ATG: autophagy-related gene; LXA4: lipoxin A4; MAPK: mitogen-activated protein kinase; NF-κB: nuclear factor kappa-B; STAT1: Signal Transducers and Activators of Transcription-1; TLR4: toll-like receptor 4; TRAF6: tumor necrosis factor receptor-associated factor-6.

It is well known that the signaling pathway of TGF-β can play an important role in fibrogenesis and also promotes fibrosis in DKD (81). Additionally, the signaling pathway of TGF-β/Smad also affects DKD through cross-talk with other pathways, such as the MAPK and PI3K/Akt signaling pathways (82). The TGF-β/Smad signaling pathway is notably activated in renal fibrosis. Smad3 exhibits a marked collagen deposition and contributes to the progression of renal fibrosis in db/db mice, and Smad3 knockout inhibits this process (83). Nagaishi et al. (39) reported that MSC-CM inhibits tubulointerstitial fibrosis in a model of DKD by reversing the endogenously elevated or ectopically expressed TGF-β. Furthermore, Bai et al. (36) demonstrated that MSC-CM reversed DKD *via* LXA4 by targeting the TGF-β/Smad pathway and pro-inflammatory cytokines. Liu et al. (84) found that MSCs modified with angiotensin-converting enzyme 2 (ACE2) to target the damaged kidney and enhance the expression of ACE2. The modified MSCs secreted soluble ACE2 protein into the culture medium. The upregulated ACE2 can degrade the Ang II into the Ang1-7, and MSCs-ACE2 is more beneficial when compared with MSCs alone in downregulating Ang II and upregulating Ang1-7. MSCs-ACE2 inhibited the deleterious effects of the accumulation of Ang II and inhibited the TGF-β/Smad pathway. Li et al. (34) found that MSC-CM alleviated renal fibrosis in a DKD model by blocking myofibroblast transdifferentiation mediated by the signaling pathway of TGF-β1/Smad2/3. MSC-CM also inhibited the proliferation of mesangial cell mediated by signaling pathways of PI3K/Akt and MAPK, and enhanced the expression of MMPs. Multiple investigations have indicated that MSC-EVs improve renal fibrosis which mediated TGF-β pathway and PI3K/Akt and MAPK signaling pathway by inhibiting the expression of TGF-β. (**Figure 3**).

## Conclusion

The importance of DKD has been increasingly acknowledged and treatment methods have been updated and improved. However, in the past 20 years, the incidence and prognosis of DKD have not been effectively controlled. MSC-based therapy brings prospective treatment to DKD. Various growth factors and miRNAs contained in MSC-EVs seem to play an important role in the therapeutic effect of MSCs. Some studies have pointed out that MSC-EVs not only protect podocytes, renal tubular epithelial cells and mesangial cells from HG-induced injury, but also protect against DKD in animals. Moreover, some studies have shown that the treatment efficacy of ACE2-modified MSCs is more effective than MSCs alone, and MSCs combined with miRNAs treatment can enhance the protective effect of MSCs. At the same time, MSC-EVs also alleviated the DKD in animals through various regulatory signaling pathways (NF-κB, TLR, mTOR, MAPK, PI3K/Akt, TGF-β/Smad). However, it is difficult to find a unified standard for the treatment of DKD with MSC extracellular vesicles. Different stem cell-derived extracellular vesicles, injection doses and frequencies are used in different experiments. Therefore, more research is needed to optimize the different MSC products, injection frequencies and doses to treat DKD.

## Author contributions

YDL and TZ looked up a lot of pertinent papers and wrote the manuscript. QY, JW, XC and YPL reviewed and checked the article. TZ modified and polished the article, and reviewed the article. All authors contributed to the article and approved the submitted version.

## Funding

This review was supported by Shantou Science and Technology Project (Shanfuke [2019] 106-4: 190606165268433), Guangdong Province Science and Technology Special Fund (shanfuke [2021]88-28: 210714086900312) and Shantou Youth Talent Project (no. 2020023).

## Conflict of interest

The authors declare that the research was conducted in the absence of any commercial or financial relationships that could be construed as a potential conflict of interest.

## Publisher’s note

All claims expressed in this article are solely those of the authors and do not necessarily represent those of their affiliated organizations, or those of the publisher, the editors and the reviewers. Any product that may be evaluated in this article, or claim that may be made by its manufacturer, is not guaranteed or endorsed by the publisher.

## Glossary

**Table:**

ACE2	angiotensin-converting enzyme 2
AD-MSCs	adipose-derived MSCs
AGEs	advanced glycation end products
Arg1	arginase-1
BM-MSCs	bone marrow-derived MSCs
BMP-7	bone morphogenetic protein-7
CCL2	C-C motif chemokine ligand 2
CHIP	Carboxyl terminus of HSP70 interacting protein
DKD	diabetic kidney disease
ECM	extracellular matrix
EGF	epidermal growth factor
EMT	epithelial-mesenchymal transition
ESRD	end-stage chronic kidney disease
ET-1	endothelin-1
EVs	extracellular vesicles
Exos	exosomes
GDNF	glial cell-derived neurotrophic growth factor
HDAC1	histone deacetylase 1
HG	high glucose
HGF	hepatocyte growth factor
HLSC	human liver stem-like cells
HRGECs	human renal glomerular endothelial cell lines
HU-MSCs	human urinederived MSCs
ICAM-1	intracellular adhesion molecule-1
LXA4	lipoxin A4
MAPK	mitogen-activated protein kinase
MCP-1	monocyte chemoattractant protein-1
MMP3	metalloproteinase 3
MSCs	mesenchymal stromal cells
MVs	microvesicles
MSC-Exos	MSC exosomes
NF-kB	nuclear factor kappa-B
PDK4	Pyruvate dehydrogenase kinase 4
PTECs	proximal tubular epithelial cells
RTEC	renal tubular epithelial cells
ROS	reactive oxygen species
STAT1	Signal Transducers and Activators of
